# Design and Synthesis of a Series of Pyrido[2,3-d]pyrimidine Derivatives as CCR4 Antagonists

**DOI:** 10.3390/molecules17089961

**Published:** 2012-08-20

**Authors:** Hongwei Gong, Hui Qi, Wei Sun, Yang Zhang, Dan Jiang, Junhai Xiao, Xiaohong Yang, Ying Wang, Song Li

**Affiliations:** 1 School of Pharmaceutical Sciences, Jilin University, Changchun 130021, China; Email: gonghw@jlu.edu.cn (H.G.); wsun@jlu.edu.cn (W.S.); 2 Laboratory of Computer-Aided Drug Design & Discovery, Beijing Institute of Pharmacology & Toxicology, Beijing 100850, China; Email: jiangdan@263.net.cn (D.J.); lis@nic.bmi.ac.cn (S.L.); 3 School of Public Health, Jilin University, Changchun 130021, China; 4 Department of Immunology, School of Basic Medical Sciences, Key Laboratory of Medical Immunology, Ministry of Health, Peking University Health Science Center, Beijing 100191, China; Email: qh20021983@163.com (H.Q.); byzhangyang08@163.com (Y.Z.); yw@bjmu.edu.cn (Y.W.)

**Keywords:** CC chemokine receptor 4 (CCR4) antagonists, CKLF1, TARC, MDC, inflammatory disease

## Abstract

A series of pyrido[2,3-d]pyrimidine derivatives were designed and synthesized based on known CC chemokine receptor 4 (CCR4) antagonists. The activities of all the newly synthesized compounds were evaluated using a chemotaxis inhibition assay. Compound **6b** was proven to be a potent CCR4 antagonist that can block cell chemotaxis induced by macrophage-derived chemokine (MDC), thymus and activation regulated chemokine (TARC), and CKLF1, the natural ligands of CCR4. In addition, compound **6b** is more effective than budesonide in the murine rhinitis model. The intravenous injection LD_50_ of compound **6b** is 175 mg/kg and the oral LD_50_ is greater than 2,000 mg/kg.

## 1. Introduction

CC chemokine receptor 4 (CCR4) is a pivotal factor in the development of allergic inflammations, such as asthma, dermatitis, and rhinitis [1]. It consists of a seven-transmembrane G-protein-coupled receptor that is selectively expressed on Th2 cell membranes. CCR4 has three natural ligands: (1) thymus and activation regulated chemokine (TARC/CCL17); (2) macrophage-derived chemokine (MDC/CCL22); and (3) chemokine-like factor 1 (CKLF1) [2,3,4]. Through the chemotaxis of the three ligands of CCR4, the Th2 cells are attracted to the sites of allergic inflammation. The number of MDC, TARC, and CCR4-expressing T cells is increased in asthmatic lungs and airways. In murine asthmatic models, the CCR4 blocking antibody attenuates airway eosinophilia and goblet cell hyperplasia and diminishes IgE synthesis and bronchial hyperreactivity [5]. Like the CCR4 antibody, the special Ab against TARC and MDC can also reduce airway eosinophilia and hyperresponsiveness in asthmatic mice elicited by OVA [6,7]. Furthermore, CCR4 knockout mice treated with *Mycobacterium bovis* Bacille–Calmette–Guerin experience a two-week delay in bacterial clearance and diminished late-stage inflammation [8]. Therefore, CCR4 and its three ligands (TARC, MDC, and CKLF1) play important roles in allergic inflammations, and CCR4 antagonists have a huge potential in the therapeutics of the allergic diseases. In addition, interrupting the interaction between CCR4 and its ligands is a potential therapeutic route for autoimmune diseases.

CKLF1 is the third natural ligand of CCR4. Although it bears no significant similarity to TARC and MDC, there are some same pivotal amino acids beside the conserved CC motif [4]. Mice with overexpressed CKLF1 have significant pathological changes that are similar to those of asthma, such as peribronchial leukocyte infiltration, tracheal epithelial shedding, and collagen deposition in lungs. Obvious pathological changes also appeared in the lungs of the CKLF1 transgenic mice, whereas no such change was observed in other organs [9]. Interestingly, the CKLF1 C-terminal peptides C19 can inhibit chemotaxis induced by both CKLF1 and TARC. In the asthmatic mouse model, C19 can reduce airway eosinophilia, lung inflammation, and airway hyperresponsiveness. However, the CKLF1 C-terminal peptide C27 has the same functional activity as that of CKLF1 [10].

As the studies on CCR4 deepen, an increasing number of highly active small molecular CCR4 antagonist classes have been discovered [11,12,13,14,15,16,17,18,19,20]. All the CCR4 antagonists are inhibitors of TARC and MDC. Our research aimed to develop more potent CCR4 antagonists that can inhibit the emigration of CCR4-expresing cells induced by MDC, TARC, and CKLF1, so a series of pyrido[2,3-d]pyrimidine derivatives were designed and synthesized, and the activities of all the newly synthesized compounds were evaluated using a chemotaxis inhibition assay.

## 2. Results and Discussion

### 2.1. Chemistry

**Figure 1:**
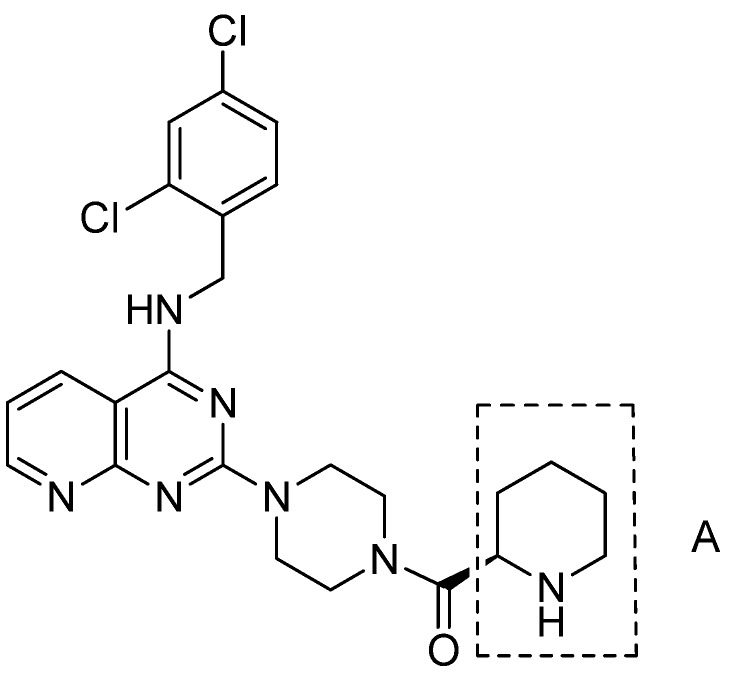
The structure of BMS-397.

**Scheme 1:**
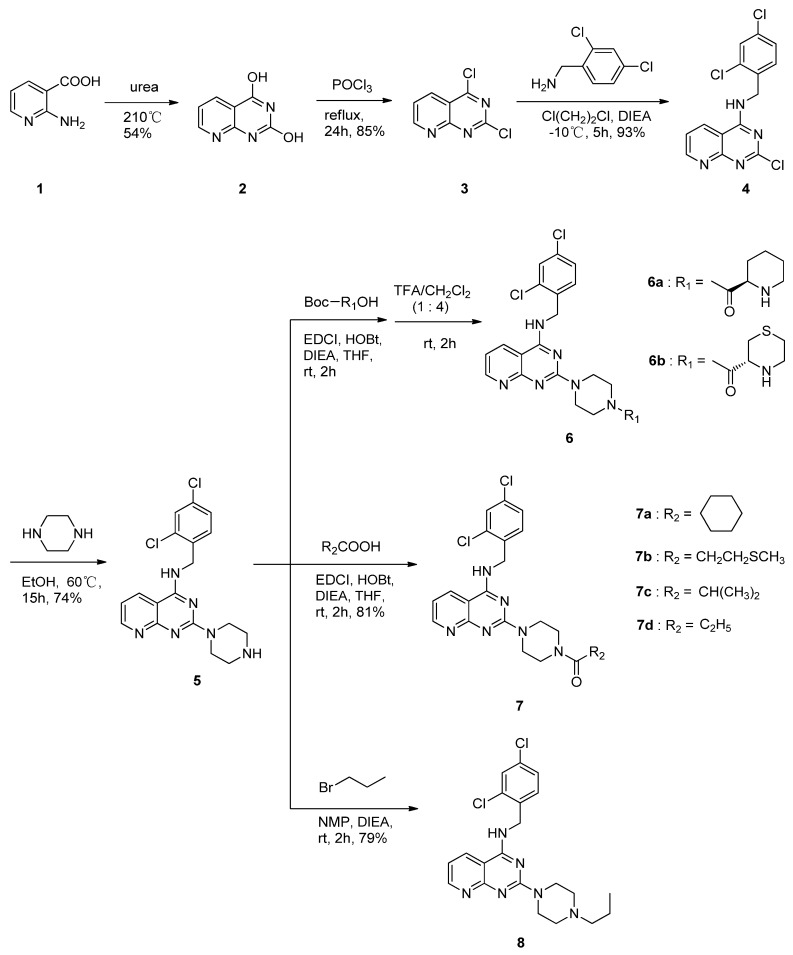
Synthesis of **6a** and **6b**, **7a**–**d**, and **8**.

Compound BMS-397 (Figure 1) is the most potent CCR4 antagonist for TARC and MDC among all the antagonists [11]. By researching the structure-activity realationship of BMS-397, we presumed that the section A of BMS-397 has large contribution to the activity. This led us to modify this site by introducing different lengths of carbochains and carbocycles, including heteroatoms. Following the design, **6b**, **7a**–**d** and **8** have been synthesized, and the synthetic routes are illustrated in Scheme 1. According to well-established literature procedures, the thermal cyclization of commercially available 2-aminonicotinic acid and urea produced **2**, which was further chlorinated with phosphorus oxychloride to produce **3** [21]. Intermediate **3** was sequentially nucleophilically substituted with 2,4-dichlorobenzylamine and piperazine to produce **5**. Then, **5** was condensed with (*R*)-1-(*tert*-butoxy-carbonyl)piperidine-2-carboxylic acid or (*R*)-4-(*tert*-butoxycarbonyl)thiomorpholine-3-carboxylic acid to produce the corresponding amides. Thereafter, Boc deprotection with trifluoroacetic acid and dichloromethane was performed to produce the desired compounds **6a** and **6b**, respectively. Finally, compounds **7a** to **7d** were synthesized by the condensation reaction of **5** with different carboxylic acids (R_2_COOH), and compound **8** was then obtained by the nucleophilic substitution of **5** and bromopropane. All of the synthesized compounds were characterized using ^1^H-NMR and MS analyses.

### 2.2. Chemotaxis Inhibition Assay

The activities of all the synthesized compounds were evaluated using a CCR4-MDC/TARC/C27 chemotaxis inhibition assay (Table 1). Considering that the functional activity of CKLF1-C27 is similar to that of CKLF1, we performed a chemotaxis inhibition assay induced by C27 [10,22]. Compound **6a** (BMS-397) was used as the calibration or comparison standard for the results in all the assays, because it is one of the most potent CCR4 antagonists [11]. In Table 1, compound **6b** demonstrate proximate chemotaxis inhibition activities for MDC/TARC and better chemotaxis inhibition activities for C27 compared with compound BMS-397.

According to biological evaluation results (Table 1), the introduction of carbonyl groups interacting with piperazine increases inhibitory potency, because **7d** exhibits a higher activity than **8**. The changes from **7a**, **7b**, to **7c**, **7d** demonstrate that the CCR4-MDC/TARC chemotaxis inhibitory potency is enhanced by increasing the volume of the groups interacting with carbonyl. Furthermore, the cyclic groups with hydrogen bond donors exhibit the best contribution to the activity, because both **6a** and **6b** are more effective than **7a**. However, for the CCR4-C27 chemotaxis inhibition, the structure–activity relationship follows an opposite rule (*i.e.*, **7a** with a larger substitute exhibits a lower inhibitory activity than **7b**, **7c** and **7d** for CCR4-C27).

**Table 1:** Chemotaxis inhibition assay results for compounds(Inh. % at 1μM).

Compound	BMS-397	6b	7a	7b	7c	7d	8
CCR4-MDC	60.3 ± 3.5	61.4 ± 1.0	52.2 ± 4.3	40.0 ± 4.9	32.0 ± 0.3	29.7 ± 3.7	23.3 ± 6.7
CCR4-TARC	59.2 ± 4.0	57.3 ± 4.7	48.8 ± 5.4	47.7 ± 7.2	48.7 ± 6.3	48.1 ± 6.6	34.8 ± 0.6
CCR4-C27	9.6 ± 3.5	21.4 ± 1.5	16.8 ± 5.2	35.7 ± 7.0	34.7 ± 0.7	37.1 ± 1.8	−1.5 ± 8.8
CCR3-CCL11		2.9 ± 8.9					
CCR5-CCL5		−7.4 ± 41.4					
CXCR1-IL-8		2.9 ± 5.1					
CXCR4-SDF-1		−4.8 ± 3.2					

^a^ Values are means of three independent experiments.

Compound **6b** demonstrates high chemotaxis inhibition activities for CCR4. In order to study the receptor specificity of compound 6b, we evaluated its chemotaxis inhibition activities for the related chemokine receptors, CCR3, CCR5, CXCR1 and CXCR4 (Table 1). As shown in Table 1, the poor chemotaxis inhibition activities proved that compound **6b** has high selectivity when tested against the related chemokine receptors (CCR3, CCR5, CXCR1 and CXCR4).

### 2.3. Effects of ***6b*** Administration on Symptoms of Murine Allergic Rhinitis

In the murine rhinitis model (sensitized with ovalbumin), budesonide (an efficient glucocorticoid) was used as the calibration or comparison standard to assess the relative efficacy of the compound. Five parameters was used to assess the effects of compounds administration on symptoms of murine allergic rhinitis: (1) the number of sneezing in ten minutes; (2) the number of rubbing nose in ten minutes; (3) the IL-4 level in the bronchoalveolar lavage fluid; (4) the IgE level of serum; (5) the number of eosinophils in noses and pulmonary tissues [23]. The efficacy of 1.28 mg/Kg of budesonide in the five parameters was achieved by only 10 μg/Kg of compound **6b** (data not published).

### 2.4. Determination of Acute Toxicity

The acute toxicity of compound **6b** was determined with up-and-down procedure. The intravenous injection LD_50_ of compound **6b** in female Kunming mice is 175 mg/kg and the oral LD_50_ is greater than 2,000 mg/kg. The results indicate that compound **6b** has low bioavailability and the security is poor. Considering the administration dose is only 10 μg/Kg, the therapeutic window is very wide.

## 3. Experimental

### 3.1. Chemistry

#### 3.1.1. Materials and Reagents

Melting points were determined using a YRT-3 melting point detector and were uncorrected. The NMR spectra were recorded using a Bruker ARX 400 spectrometer (Karlsruhe, Germany). The mass spectra were determined using an Agilent 5875(EI) spectrometer (Palo Alto, CA, USA). All solvents and reagents were purchased commercially and used without further purification.

#### 3.1.2. Chemical Synthesis

*Pyrido[2,3-d]pyrimidine-2,4-diol* (**2**). Compound **2** was synthesized according to a well-established literature procedure [21]. Yield 54%. ^1^H-NMR (DMSO-*d*_6_) δ ppm: 11.69 (1H, s), 11.48 (1H, s), 8.61 (1H, m), 8.27 (1H, m), 7.26 (1H, m).

*2,4-Dichloropyrido[2,3-d]pyrimidine* (**3**). Compound **3** was synthesized according to a well-established literature procedure [21]. Yield 85%. ^1^H-NMR (CDCl_3_) δ ppm: 9.34 (1H, m), 8.66 (1H, m), 7.76 (1H, m); EI-MS (*m/z*): 199.0 [M]^+^.

*2-Chloro-N-(2,4-dichlorobenzyl)pyrido[2,3-d]pyrimidin-4-amine* (**4**). 2,4-Dichlorobenzylamine (10.03 g, 0.057 mol) was dropped into the mixture of compound **3** (10.36 g, 0.052 mol) and *N*,*N*-diisopropylethylamine (DIEA, 7.35 g, 0.057 mol) in 1,2-dichloroethane (90 mL) under −10 °C. After stirring for 5 h, the precipitate was filtered to obtain compound 4 (16.35 g, 93%) as a white solid. ^1^H-NMR (DMSO-*d*_6_) δ ppm: 9.57 (1H, m), 9.03 (1H, m), 8.79 (1H, m), 7.69 (1H, m), 7.64 (1H, m), 7.45 (2H, m), 4.79 (2H, d, *J* = 5.2 Hz); EI-MS (*m/z*): 339.2 [M+H]^+^.

*N-(2,4-Dichlorobenzyl)-2-(piperazin-1-yl)pyrido[2,3-d]pyrimidin-4-amine* (**5**). A mixture of compound **4** (16.35 g, 0.048 mol) and piperazine (8.27 g, 0.096 mol) in ethanol (1,200 mL) was heated to 60 °C and stirred for 15 h. Ethanol was removed under reduced pressure. The residue was purified through column chromatography (silica gel) eluted with ethyl acetate, methanol, and ammonia water (v:v:v = 1:1:0.039) to obtain compound 5 (13.86 g, 74%) as a white solid. ^1^H-NMR (DMSO) δ ppm: 8.85 (1H, m), 8.66 (1H, m), 8.46 (1H, m), 7.63 (1H, m), 7.38 (2H, m), 7.10 (1H, m), 4.73 (2H, d, *J* = 5.2 Hz), 3.63 (4H, s), 2.61 (4H, s); EI-MS (*m/z*): 389.2 [M+H]^+^.

*(R)-(4-(4-((2,4-dichlorobenzyl)amino)pyrido[2,3-d]pyrimidin-2-yl)piperazin-1-yl) (thiomorpholin-3-yl)methanone* (**6b**). A mixture of compound **5** (1.28 g, 3.28 mmol), (*R*)-4-(*tert-*butoxycarbonyl)thiomorpholine-3-carboxylic acid (0.81 g, 3.28 mmol), 1-(3-dimethylaminopropyl)-3-ethylcarbodiimide hydrochloride (EDCI) (0.94 g, 4.92 mmol), 1-hydroxybenzotriazole (HOBt) (0.66 g, 4.92 mmol), and DIEA (0.85 g, 6.56 mmol) in tetrahydrofuran (40 mL) was stirred for 2 h at room temperature. The solvent was removed under reduced pressure. 20 mL of trifluoroacetic acid and dichloromethane (v:v = 1:4) were added to the residue, and the mixture was stirred for 2 h at room temperature. The solvent was removed under reduced pressure. Then, 20 mL of water was added to the residue. The pH of the solution was raised to 8 with 1 N sodium hydroxide, and the solution was extracted using dichloromethane (3 × 20 mL). The combined dichloromethane extracts were concentrated under reduced pressure. Thereafter, the residue was purified through column chromatography (silica gel) eluted with ethyl acetate, methanol, and ammonia water (v:v:v = 6:1.5:0.039) to produce compound **6b** (1.23 g, 73%) as a white solid, m.p.: 168–170 °C. ^1^H-NMR (CDCl_3_) δ ppm: 8.79 (1H, m), 7.93 (1H, dd, *J* = 2.0 Hz, *J* = 1.7 Hz), 7.44 (1H, d, *J* = 2.0 Hz), 7.34 (1H, d, *J* = 8.4 Hz), 7.22 (1H, m), 7.05 (1H, m), 6.26 (1H, t), 4.86 (2H, d, *J* = 5.9 Hz), 4.09–3.44 (10H, brm), 3.14 (1H, m), 2.82 (2H, m), 2.44 (2H, m). EI-MS (*m/z*): 518.3 [M+H]^+^. Elemental analysis, calculated for C_23_H_25_Cl_2_N_7_OS (518.46): C, 53.28; H, 4.86; N, 18.91. Found: C, 52.99; H, 4.84; N, 18.86.

*Cyclohexyl(4-(4-((2,4-dichlorobenzyl)amino)pyrido[2,3-d]pyrimidin-2-yl) piperazin-1-yl) methan-one* (**7a**). A mixture of compound **5** (0.4 g, 1.03 mmol), cyclohexanecarboxylic acid (0.13 g, 1.03 mmol), EDCI (0.30 g, 1.55 mmol), HOBt (0.21 g, 1.55 mmol), and DIEA (0.27 g, 2.06 mmol) in tetrahydrofuran (10 mL) was stirred for 2 h at room temperature. The solvent was removed under reduced pressure. Then, the residue was purified through column chromatography (silica gel) eluted with ethyl acetate, methanol, and ammonia water (v:v:v = 15:1:0.078) to obtain compound **7a** (0.41 g, 81%) as a white solid, m.p.: 177–179 °C. ^1^H-NMR (CDCl_3_) δ ppm: 8.76 (1H, m), 8.05 (1H, d, *J* = 7.0 Hz), 7.41 (1H, d, *J* = 2.0 Hz), 7.35 (1H, d, *J* = 8.1 Hz), 7.21 (1H, m), 7.04 (1H, m), 6.56 (1H, s), 4.86 (2H, d, *J =* 5.9 Hz), 3.96 (4H, d, *J =* 20.8 Hz), 3.65 (2H, s), 3.52 (2H, s), 2.51 (1H, m), 1.83 (5H, m), 1.52 (2H, m), 1.28 (3H, m); EI-MS (*m/z*): 499.1 [M+H]^+^. Elemental analysis, calculated for C_25_H_28_Cl_2_N_6_O (499.44): C, 60.12; H, 5.65; N, 16.83. Found: C, 59.91; H, 5.62; N, 16.79.

*1-(4-(4-((2,4-Dichlorobenzyl)amino)pyrido[2,3-d]pyrimidin-2-yl)piperazin-1-yl)-3-(methylthio)- propan-one* (**7b**), *1-(4-(4-((2,4-Dichlorobenzyl)amino)pyrido[2,3-d]pyrimidin-2-yl)-piperazin-1-yl)-2-methylpropan-1-one* (**7c**) and *1-(4-(4-((2,4-dichlorobenzyl)amino)pyrido[2,3-d]-pyrimidin-2-yl)piperazin-1-yl)propan-1-one* (**7d**). The compounds **7b**, **7c** and **7d** were obtained as white solids through the same method used to obtain compound **7a**. Compound **7b**, m.p.: 201–203 °C. ^1^H-NMR (CDCl_3_) δ ppm: 8.75 (1H, m), 8.10 (1H, d, *J* = 7.6 Hz), 7.41 (1H, d, *J* = 2.0 Hz), 7.34 (1H, d, *J* = 8.0 Hz), 7.20 (1H, m), 7.05 (1H, m), 6.71 (1H, s), 4.85 (2H, d, *J* = 6.4 Hz), 3.91 (4H, brm), 3.67 (2H, m), 3.51 (2H, m), 2.86 (2H, t, *J* = 7.3 Hz, *J* = 7.8 Hz), 2.69 (2H, t, *J* = 7.8 Hz, *J* = 7.0 Hz), 2.15 (3H, s); EI-MS (m/z): 495.4 [M+H]^+^. Elemental analysis, calculated for C_22_H_24_Cl_2_N_6_OS (491.44): C, 53.77; H, 4.92; N, 17.10. Found: C, 53.69; H, 4.88; N, 17.03. Compound **7c**, m.p.: 218–219 °C. ^1^H-NMR (CDCl_3_) δppm: 8.77 (1H, m), 8.02 (1H, d, *J* = 7.6 Hz), 7.42 (1H, d, *J* = 2.0 Hz), 7.35 (1H, d, *J* = 8.2 Hz), 7.21 (1H, m), 7.05 (1H, m), 6.49 (1H, s), 4.86 (2H, d, *J* = 5.6 Hz), 3.98 (4H, d, *J* = 21.6 Hz), 3.66 (2H, m), 3.54 (2H, m), 2.86 (1H, m), 1.17 (6H, d, *J* = 6.7 Hz); EI-MS (m/z): 459.2[M+H]^+^. Elemental analysis, calculated for C_22_H_24_Cl_2_N_6_O (459.37): C, 57.52; H, 5.27; N, 18.29. Found: C, 57.51; H, 5.26; N, 18.27. Compound **7d**, m.p.: 209–210 °C. ^1^H-NMR (CDCl_3_) δ ppm: 8.76 (1H, m), 8.07 (1H, d, *J* = 7.3 Hz), 7.42 (1H, d, *J* = 2.2 Hz), 7.35 (1H, d, *J* = 8.4 Hz), 7.2 (1H, m), 7.05 (1H, m), 6.61 (1H, s), 4.86 (2H, d, *J* = 5.9 Hz), 3.97 (4H, d, *J* = 19.3 Hz), 3.66 (2H, m), 3.50 (2H, m), 2.43 (2H, q), 1.20 (3H, t); EI-MS (m/z): 444.9 [M+H]^+^. Elemental analysis, calculated for C_21_H_22_Cl_2_N_6_O (445.34): C, 56.64; H, 4.98; N, 18.87. Found: C, 56.64; H, 4.99; N, 18.86.

*N-(2,4-Dichlorobenzyl)-2-(4-propylpiperazin-1-yl)pyrido[2,3-d]pyrimidin-4-amine* (**8**). A solution of compound **5** (0.4 g, 1.03 mmol), 1-bromopropane (0.14 g, 1.13 mmol), and DIEA (0.15 g, 1.13 mmol) in 1-Methyl-2-pyrrolidone (NMP, 10 mL) was stirred for 2 h at room temperature. The solvent was removed under reduced pressure. The residue was then purified through column chromatography (silica gel) eluted with ethyl acetate, methanol, and ammonia water (v:v:v = 15:1:0.078) to obtain compound 8 (0.35 g, 79%) as a white solid, m.p.: 181–182 °C. ^1^H-NMR (CDCl_3_) δ ppm: 8.74 (1H, m), 7.90 (1H, d, *J =* 6.7 Hz), 7.41 (1H, d, *J =* 2.2 Hz), 7.36 (1H, d, *J =* 8.1 Hz), 7.21 (1H, m), 6.99 (1H, m), 6.18 (1H, s), 4.86 (2H, d, *J =* 5.6 Hz), 3.97 (4H, s), 2.48 (4H, s), 2.36 (2H, t, *J =* 7.6 Hz, *J =* 7.8 Hz), 1.57 (2H, m), 0.95 (3H, t, *J =* 7.3 Hz, *J =* 7.6 Hz); EI-MS (*m/z*): 431.2 [M+H]^+^. Elemental analysis, calculated for C_21_H_24_Cl_2_N_6_ (431.36): C, 58.47; H, 5.61; N, 18.48. Found: C, 58.25; H, 5.62; N, 18.45.

### 3.2. Bioassay Methods for Chemotaxis Inhibition

The chemotaxis assay was performed using a 48-well microchemotaxis chamber (Neutroprobe, Bethesda, MD , USA). C27 (Hybio Engineering Company, Shenzhen, China)/hCCL17/hCCL22 (Peproteche, Rocky Hill, NJ, USA) was diluted using buffer (RPMI 1640, 0.1% BSA) to a 100 ng∙mL^−1^/80 ng∙mL^−1^/10 ng∙mL^−1^ final concentration and placed in the lower wells (27.5 µL/well). The compounds were diluted with DMSO to achieve a 1 mM concentration and then diluted to a 10 µM concentration in 0.1% BSA medium. The HEK293 cells that were transfected with pcDI-CCR4 were suspended in an assay buffer at 1 × 10^6^ cells/mL, incubated with the compounds (1 µM final concentration) for 30 min at 37 °C, and then added to the upper wells (55 µL/well). Between the lower and upper wells are polyvinylpyrrolidone-free polycarbonate filters (10 µm pores, Neutroprobe) coated with Rat Tail Collagen Type 1 (Biomedical Technologies). The chamber was incubated for 5 h at 37 °C with 5% CO_2_. The filters were removed from the chamber, washed, fixed, and stained using the Three Step Stain Set (Richard-Allen Scientiﬁc Michigan, MI, USA). The migrated cells were counted in five randomly selected high-power fields (400×) per well. All samples were assayed thrice. The chemotactic index (CI) is the ratio of the number of cells that migrated to the sample to the number of cells that migrated to the 0.1% BSA medium. The standard for the significant chemotaxis is CI > 2. The chemotaxis inhibition percentage was computed using the formula (1 − CI_compound-pretreated cells_/CI_DMSO-pretreated cells_) × 100%. The chemotaxis assay of compound **6b** for receptors, CCR3, CCR5, CXCR1, CXCR4 was performed using the same method with CCR4 chemotaxis inhibition. The HEK293 cells were transfected with pcDI-CCR3/CCR5/CXCR1/CXCR4, and the concentration of CCL11/ CCL5/ IL-8/ SDF-1 is 100 ng∙mL^−1^.

## 4. Conclusions

In summary, we designed and synthesized a series of pyrido[2,3-d]pyrimidine derivatives and evaluated their *in vitro* and *in vivo* activities. Compound **6b** was found to be a potent CCR4 antagonist (in the CCR4-MDC/TARC/C27 chemotaxis inhibition assay). The current study also proved that compound **6b** is even more effective than budesonide in the murine rhinitis model. Although the intravenous injection LD_50_ of compound **6b** is low (175 mg/kg), the therapeutic window is very wide. Further studies on the modification and mechanism of these compounds are in progress.
